# The role of emotions in cancer patients’ decision-making

**DOI:** 10.3332/ecancer.2019.914

**Published:** 2019-03-28

**Authors:** Ketti Mazzocco, Marianna Masiero, Maria Chiara Carriero, Gabriella Pravettoni

**Affiliations:** 1 Department of Oncology and Hemato-oncology, University of Milan, 20122 Milan, Italy; 2 Applied Research Division for Cognitive and Psychological Science, European Institute of Oncology IRCCS, 20141 Milan, Italy; 3 Department of Biomedical and Clinical Sciences, University of Milan, 20122 Milan, Italy

**Keywords:** emotion, anxiety, fear, worry, cancer, health behaviour, patient’s decision making

## Abstract

**Introduction:**

Despite the attempt to make decisions based on evidence, doctors still have to consider patients’ choices which often involve other factors. In particular, emotions seem to influence the way that options and the surrounding information are interpreted and used.

**Objective:**

The objective of the present review is to provide a brief overview of research on decision making and cancer with a specific focus on the role of emotions.

**Method:**

Thirty-nine studies were identified and analysed. Most of the studies investigated anxiety and fear. Worry was the other psychological factor that, together with anxiety, played a crucial role in cancer-related decision-making.

**Results:**

The roles of fear, anxiety and worry were described for detection behaviour, diagnosis, choice about prevention and curative treatments and help-seeking behaviour. Results were inconsistent among the studies. Results stressed that cognitive appraisal and emotional arousal (emotion’s intensity level) interact in shaping the decision. Moderate levels of anxiety and worry improved decision-making, while low and high levels tended to have no effect or a hindering effect on decision making. Moderating factors played an under-investigated role.

**Conclusions:**

Decision making is a complex non-linear process that is affected by several factors, such as, for example, personal knowledge, past experiences, individual differences and certainly emotions. Research studies should investigate further potential moderators of the effect of emotions on cancer-related choice. Big data and machine learning could be a good opportunity to test the interaction between a large amount of factors that is not feasible in traditional research. New technologies such as eHealth and virtual reality can offer support for the regulation of emotions and decision making.

## Introduction

Cancer remains a worldwide leading cause of death and constitutes a significant burden to patients and their families, despite the fact that cancer mortality rates are estimated to decline as a result of increased emphasis on early detection, improved treatment methods and the adoption of healthy lifestyles [1, 2]. A cancer diagnosis remains a life time-point where the person has to make choices regarding available treatments and actions to be taken to face the care pathway, other than life changes.

Despite the attempt to make decisions based on evidence, studies in psychology, economics and also in the medical field show that often emotions prevail, affecting the thinking process and therefore the choice [3–6].

Decision-making has acquired a crucial importance in cancer care in the last 20 years, since the introduction of the patient-centred approach which emphasises the new relationship between patients and doctors and the increasingly common model of shared decision-making. Patients are not passive recipients anymore but active participants of the care process. In this vein, patients are required to take several decisions during all phases of the disease. Despite the doctor’s efforts to provide the patient with all necessary objective information for reaching a good shared decision, often the patient’s choice is affected by other factors than objective information, emotion being among the most relevant. Fear of surgery or of possible side effects might delay or stop the decision to undergo specific clinical procedures or treatments [7]. Particularly, in life-threatening health conditions such as cancer, evidence shows that emotions such as fear and anxiety affect the choice [8]. For example, fear of adverse events leads most patients to refuse participation in clinical trials [9]. Negative emotions elicited by the new (negative) situation often means that attention is focused on the negative aspects [10], producing a distortion in risk perception and, consequently, suboptimal health decisions [3]. For example, information presented during the first consultation is often forgotten or very difficult to memorise because of patients’ emotional state [11, 12]. Also, smokers tend to postpone the decision to quit smoking because of the positive visceral emotions they feel (e.g. hedonistic pleasure related to smoking) or because of the attribution of the disease to uncontrollable factors (e.g., genetic predisposition, environmental pollution and so on) in order to cope with fear [13]. Emotions, as well as cognition, are a fundamental component of the decision-making process.

### Emotions

A definition of the term ‘emotion’ is not so easy, as Izard showed in his interview with scientists working on emotions [14]. ‘Emotion consists of neural circuits (that are at least partially dedicated), response systems and a feeling state/process that motivates and organises cognition and action. Emotion also provides information to the person experiencing it and may include antecedent cognitive appraisals and ongoing cognition, including an interpretation of its feeling state, expressions or social-communicative signals and may motivate approach or avoidant behaviour, exercise control/regulation of responses and be social or relational in nature’ [14, p 367]. The complexity of emotion can be explained by its multi-aspect nature.

The main approaches in the study of the structure of emotion are two: the categorical and the dimensional. While the categorical approach affirms the existence of several distinct emotions, such as fear, anger, disgust, happiness and sadness [15], the dimensional approach describes the structure of two dimensions of positive and negative affect [16] or two dimensions of valence (a continuum that varies from unpleasant to pleasant) and arousal (from calm to excited) [17].

Valence and arousal were identified as the most basic dimensions [18] that inform the evaluative appraisal (the cognitive meaning attributed to the emotion). The importance of appraisal in the experience of emotion can find its root in the theories of Cannon [19, 20] and Bard [21]: in order to be experienced as emotion, the emotion needs a cognitive evaluation to be associated with physiological changes (arousal). This theoretical position was developed further by the appraisal theories that foster the importance of the cognitive evaluation of the event. Appraisal consists of the rapid and often unconscious evaluation of a stimulus or an event, and that eventually determines and differentiates the emotions felt subjectively [22], by using information from the event in its context and from the individual’s mental map (e.g. personal knowledge, beliefs, history) [23]. The criteria for the event evaluation on which most theoreticians agree are those of novelty, intrinsic pleasantness, predictability, goal-relevance, the coping potential and the compatibility with personal and social norms [24]. The high subjectivity in evaluating the event provides the reason for the large degree of variability in the characterisation of emotions [25].

The connotative meaning of an event depends on the interaction between valence and arousal. More specifically, arousal impacts on the evaluative appraisal of negative stimuli more than of positive stimuli in line with the hypothesis of negativity bias that claims a priority of high-arousing negative stimuli in the processing of affective stimuli. Studies using neuroimaging confirmed the involvement of cognitive processes in the experience of emotion [26], showing the activation of brain areas associated with attentional processes, language and long-term memory when dealing with several different emotions. The involvement of cognitive processes such as attention and perception can be bottom-up that is driven by the stimulus, or top-down when the appraisal of the situation depends on stored knowledge of similar situations [27]. Lazarus and Folkman’s [28] transactional model supports the top-down process, postulating that the cognitive assessment of an event can trigger emotions such as anxiety, sadness and anger. An example of cognitive assessment influencing emotions is the catastrophising phenomenon [29–31]. Catastrophising can be defined as ‘a tendency to magnify or exaggerate the threat value’ of an event [32, p 111] and is characterised, other than by magnification, by rumination and defencelessness [28]. According to the transactional model, catastrophising is described as a result of underlying beliefs, evaluative appraisal and the evaluation of the ability to cope with the situation. Coherently, if a person evaluates a stimulus as threatening and estimates that he or she is unable to handle the situation, this might result in catastrophising. Catastrophic interpretations are considered the cognitive precursor of emotional reactions [33, 34] and salient feature associated with hypervigilance and fear of an immediate actual threat [35, 36] or anxiety for a more vague, uncertain and future-oriented threat [37, 38]. Common aspects in the frameworks describing catastrophising are the relation with emotional responding and coping: an increased emotional state and the avoidance of prospects associated with the threat.

Besides an evaluation of valence, arousal and appraisal, emotion can also be defined considering the type of relationship between emotion itself and the object or event to be evaluated. From this perspective, emotion can be considered incidental or integral and immediate or anticipated.

*Incidental emotions* are emotions elicited by one situation (for example, a fight with the neighbour or sunny weather) and extended to another non-related context (for example, buying a new car or deciding on a new job). They are elicited by a person, a situation or by stimuli not normatively relevant to the decision. Chou *et al* [39] reported evidence on the association between mood (positive, negative or neutral) and risk-taking behaviour. They found more risk-taking behaviours in people in a happy mood than in a sad mood. Similarly, people reading a newspaper story designed to induce a negative mood tended to overestimate the probability for various potential causes of death than those who read a newspaper story designed to induce a positive mood [40].

Despite the fact that incidental emotions should not affect judgement and decisions on unrelated tasks (the type of new car to buy after a fight with a neighbour or on a sunny day), these emotions actually have been shown to shape risk perception and decisions. For example, while a patient may feel fear because of a cancer diagnosis, at the same time he/she continues to feel anger over a previous argument with his/her spouse. Despite the fact that such incidental emotion (anger over the spouse) is not normatively relevant for the choice [41], it can still be salient and meaningful and affect treatment-related information processing and decisions. This is especially true for people low in numeracy for whom numerical expressions of risk have little meaning [42].

In contrast to incidental emotions,* integral emotions* are experienced at the time of the decision in response to a new or threatening situation [43]. For example, the fear someone feels when a bear suddenly appears in front of him/her, the anxiety a person feels about the potential outcome of a risky choice [44] or the fear, anger and sadness experienced related to cancer [42]. The adaptive function is particularly evident when dealing with integral emotion: it provides information about whether the stimulus or event is relevant for the individual’s present or future survival or well-being [22] and modulates basic cognitive processes, such as attention, perception and memory, involved in information processing and stimulus evaluation [45].

Integral emotions can, in turn, be categorised into *immediate* and* anticipated emotions*.

While *immediate* emotion is an immediate visceral reaction triggered by the situation, anticipated emotion is defined as the effect that people expect they will feel once those outcomes take place [46]. *Anticipated* emotions are particularly important for decision making where the individual has to anticipate the possible consequences (including emotions) of a choice. This sort of simulation consists of pre-experiencing the event and pre-feeling the anticipated emotion and is affected by previous experiences and memories of similar events [47]. To make a choice, the decision maker anticipates the pleasure of the first vacation assuming good weather and the displeasure of the vacation assuming bad weather. In the oncological field, while making the decision whether to undergo risk-reducing mastectomy (RRM) or to adhere to a surveillance programme, a healthy woman with a BRCA mutation increasing the risk of breast cancer will anticipate emotions associated with the outcomes of the options to be selected. For example, the relief associated with the risk reduction following the RRM or the regret associated with the possible occurrence of cancer in case of the selection of a surveillance programme. These feelings are weighted by the perceived likelihood of good or bad outcomes (in our example, no cancer or occurrence of cancer), respectively, and the resulting feelings are combined to obtain an average feeling of anticipated pleasure [48].

### Emotion and decision making

As a recent review showed [44] emotions are the dominant driver of most meaningful decisions in life [46, 49–52]. Decisions can be viewed as a conduit through which emotions guide everyday attempts to avoid negative feelings (e.g. guilt and regret) and increasing positive feelings (e.g. pride and happiness), even when they do so without awareness.

However, for a long period, emotions have been excluded from the decision-making domain. *Normative* approach, promoted by Von Neuman and Morgerstern [53], affirmed that the ‘best choice’ is the choice able to maximise the subjective expected utility. In this model, the decision-maker is represented by the ‘*Homo Oeconomicus*’ completely rational, with a stable preference system able to evaluate all options and to choose the one with a higher utility. In this vein, by maximising utilities, decision makers maximise pleasure and minimise pain [48]. Accordingly, emotions are roadblocks that hinder the process of achieving an optimal decision. In the 60s, a new approach was proposed by Simon [54] and Tversky and Kahneman [55] highlighting the limits of human rationality, describing the so-called ‘*Homo Heuristicus*’. Simon’s ‘Bounded Rationality’ illustrates the psychological and contextual factors affecting judgement and decision, positing that human behaviour is determined by the ‘inner environment of people’s minds’ [54]. Two decades later, Tversky and Kahneman [55] provided another milestone with the introduction of the ‘Prospect Theory’, showing that the construction of preferences and risk perception depend on the individual’s *status quo* that influences the interpretation of the absolute value of an option. Despite the importance of these contributions, emotions are not yet explicitly considered, focus still being strongly on cognitive processes [46]. Only since the 80s, an ‘*Emotion Revolution*’ has been directed, stressing the role of affective processes in decision-making [56]. In this revolution, Loomes and Sudgen’s [57], Bell’s [58], Zajonc’s [59] and Damasio’s [60] contributions are important milestones.

Zajonc [59] argued that affective reactions to stimuli are often the very first reactions, occurring automatically and subsequently guiding information processing and judgement. This idea of emotion as a guide for judgement and decision making is strongly supported by the *somatic marker hypothesis* [60]. According to this hypothesis, emotions are the result of bodily sensations associated to specific events through experience, that eventually are translated into mental images stored in the long-term memory. When a person starts thinking about the possible consequences of a decision, emotions may be triggered by emotion-laden images stored in memory and associated with those consequences. When negative, emotions sound as an alarm. When positive, they are a signal of incentive. According to this hypothesis, automatic bodily arousal responses (somatic markers) are triggered by emotion-laden events that are consequently marked with an emotional signal. Of key importance in the use of emotions in decision making is the personal interoceptive ability that is the ability to detect subtle bodily changes: individuals who are high in interoception use such body information in decision making more than people with a low interoceptive ability [61]. The ability to detect even subtle bodily changes associated with an emotional reaction can help the person better evaluate the event.

According to these approaches, emotion helps the decision maker to assess the value of the option in three ways: (i) driving the search for information—‘*feeling as information mechanism*’ [62]; (ii) guiding the identification of salient information [63] and (iii) highlighting the relevant aspects of the situation contributing to preferences construction [64].

In particular, emotion seems beneficial when it is not possible or easy to determine which option is better from a cognitive or analytical comparison. When choice options present the same normative value or when the decision is too complex, emotions can help limit the range of information to take into account the inference actually drawn from potential infinity and the set of options among which to choose [65]. In this argument, De Sousa [66, p 276] stated that ‘emotions render salient only a tiny proportion of the available alternatives and of the conceivable relevant facts’.

### Effects of emotions

Theories of emotion and decision making argued that part of the calculation of the value of decision options should include the nature of the emotional response elicited by those options and associated potential outcomes. However, how this occurs varies depending on the decision attributes assessed and the specific personal emotional reaction [45].

Several studies [60, 67–69] showed that individuals with emotional dysfunctions tend to perform poorly compared with those who have intact emotional processes. Bechara *et al* [70, p 1294] argued that the autonomic response is used as ‘nonconscious signalling that reflects access to records of previous individual experience—specifically records shaped by reward, punishment and the emotional state that attends them’. In the same line of thinking of the positive role of emotion in decision making, regret may lead people to be conservative in their decision, helping them avoid the alternative with the worst outcome [57]. A healthy woman with BRCA mutation, anticipating the negative emotion that she might feel in case of cancer occurrence after deciding on active surveillance, might lead her to actually choose RRM in order to avoid cancer-related death. Similarly, a prostate cancer patient might prefer active surveillance instead of surgery because of the regret he anticipates thinking of the subjective unacceptable surgical side effect of sexual impotence.

On the other hand, although emotions can accurately guide behaviour, reliance on them can also be misleading. As Slovic *et al* [71, p 319] argued, ‘if it was always optimal to follow our affective and experiential instincts, there would have been no need for the rational-analytic system of thinking to have evolved and become so prominent in human affairs’. Examples of the negative impact of emotion can be find in the adoption of lifestyle behaviours. Smokers tend to overweight the visceral emotions connected with smoking (e.g., hedonistic pleasure related to the smoking’s action), with an under evaluation of risks related to cigarettes [13].

The negative effect of emotions on decision making can be explained by Mathews and MacLeod’s [10] model on information processing. According to this model, high levels of emotion lead the individual to focus on information congruent with the felt emotion, and consequently to a biased interpretation of the stimulus or the event. For example, high levels of fear, anxiety or anger induce people to focus on negatively connoted information, increasing the negative interpretation of the situation. While emotion can have a beneficial effect in the information search, as hypothesised by de Sousa [65, 66], high-intensity levels of emotion may then also impair the information search, interpretation and choice. During the first consultation with the oncologist in which a cancer diagnosis is provided, the patient’s emotional state induced by the negative emotion-laden information may lead them to forget or not memorise part of the consultation’s content [11].

## Aim

The present review was carried out to explore the role of emotion in patient’s decision-making related to cancer. The specific purpose was to identify the main factors associated with the emotion-decision-making relationship and to investigate the beneficial or hindering role in choice behaviour in order to eventually propose strategies to improve cancer patients’ decision-making [72].

## Methods

### Data sources and literature search strategy

This work offers a narrative and critical overview of the chief findings from previous qualitative and quantitative studies identified in a literature search on the relation between cancer, decision-making and emotion. The methodology approach used to treat the data was settled according to Collins *et al* criteria [72]. In particular, a qualitative approach was used, focusing on the main issues associated with the emotion-decision-making relationship. Studies published until 2017 were considered. The search was conducted using the PubMed and Ovid databases; and a set of keywords or a combination of them was used: emotion, cancer, decision and choice. Considering the main aim of this narrative review, a series of the inclusion and exclusion criteria were fixed. *Inclusion criteria:* (a) prospective and retrospective, case-control, longitudinal, cohort studies, randomised and clinical trials, systematic review, narrative review and meta-analysis; (b) studies concerning cancer patients and (c) studies published in the English language. *Exclusion criteria*: (a) studies that evaluated the role of emotions in non-oncological fields; (b) articles investigating decision making of special populations (e.g., alcoholics, drug-abusers); (c) studies that assessed the role of emotions not related to patients’ decision-making; (d) studies based on animal models and (e) letters, commentaries and editorials.

Notwithstanding the fact that our paper is not a systematic review, we have tracked all identified papers in order to better synthesise the collected evidence. According to this, a total of 311 articles were identified (207 from PubMed, 61 from Ovid), some articles were found through other sources (PsychINFO, Researchgate and Google Scholar). Thirty articles were removed because they were duplicates. Records screened were 288. After abstracts and titles screening, 224 more papers were excluded because they did not deal with decisions made by patients. Sixty-four full-text articles were assessed for eligibility. Twenty-five full-text articles were excluded because they did not match the inclusion criteria. Finally, after removal of papers that did not adhere to the inclusion/exclusion criteria, 39 papers were selected for the revision.

## Results

Among the 39 selected papers, the majority of studies were conducted in the United States of America and were related to choices regarding breast cancer screening and treatment, followed by ovarian cancer. The analysis of the selected papers led to the interpretation of emotions as facilitators or barriers of good decision-making in the oncological setting, where good refers to the ability to choose the option that increases the probability to obtain the best possible outcome.

Despite the fact that depression includes, among other symptoms, negative emotions such as sadness, depression is a complex psychological disorder that needs a more specific in-depth analysis. For this reason, depression will not be considered in this review. The pure effect of sadness has not been investigated on cancer patients’ decision-making.

The most studied emotions with respect to patients’ cancer-related decisions were fear and anxiety.

Despite the similarity in arousal that characterises fear and anxiety, we consider such emotional states as different. The main factor that can help distinguish the two emotions is the involvement of a conflict and the certainty of a threat. In particular, fear responds to an immediate threat, whereas anxiety is a future-oriented emotion [38].

Worry was included in the review, even though it cannot be considered an emotion in all respects, but rather the cognitive side of an emotion, and more specifically of anxiety [73, 74].

For each of the considered emotions, a brief definition of the emotion was provided, followed by its effects on decision making, categorising findings according to a timeline of the common patient’s care pathway, such as detection behaviour, diagnosis, prevention and curative treatments.

## Fear

### Definition

Fear, together with anxiety, is the predominant emotion in cancer patients. Fear can be defined as both a physiological arousal and a subjective experience resulting from the presence of a threat. In general, it helps patients face a risky situation, where an important loss is possible and the individual must ‘decide’ whether to fight or flee the cause of the potential loss. In oncology, the potential cause of the loss is not so univocal. The patient might experience fear when dealing with a diagnosis of cancer and its consequences or with the side effects of treatments. See Table 1 for a synthesis of the studies considering the association between fear and decision making.

### Choice on detection behaviour

Findings on fear affecting detection behaviour are not consistent. Ghahramanian *et al* [75] showed no effect of fear on the adoption of screening behaviour such as breast self-examination. On the contrary, Talbert [76] showed a positive effect of fear on compliance to breast cancer screening, while Ferrat *et al* [77] showed that fear of screening results was the main barrier to breast detection behaviour. An interesting finding was shown by Smith *et al* [78] on the interaction between fear and mental imagery. In particular, the authors showed that when a cancer-related mental imagery is negatively emotional connoted, and in particular is fear-connoted, the adoption of screening behaviour is higher. In particular, women recalling cancer-related memorable messages that evoked fear were more likely to perform detection behaviour, such as breast self-examination or mammogram. The association between mental imagery and fear did not show this effect on prevention behaviour, such as for example, the adoption of a healthy lifestyle (physical activity and healthy diet).

### Diagnosis and choice

Quinn *et al* [79] showed that both the fear resulting from cancer diagnosis and the fear related to the various aspects of clinical trials (e.g. side effects) are barriers to adherence to treatment and participation to clinical trials. In particular, in their interviews, fear was mentioned in relation to three main concepts: fear associated with cancer diagnosis; fear of clinical trials and fear of the unknown associated with cancer diagnosis, cancer treatment and clinical trials. As exemplified by some patients, the first reaction after a diagnosis of cancer is an intense fear that seems to freeze patients’ thinking, as exemplified by some participants ‘*I was so afraid. I was sure I was going to die when I found out I had cancer. It was terrifying. I could not think about anything but what I was going to miss…*’ or ‘*my family and I were so scared when the doctor said ‘cancer’; I don’t think I heard anything else that was said’.* The role of fear in making patients decide not to participate in a clinical trial can be summarised also by the following patients saying *‘It* [the clinical trial]* brings up fear, it is frightening—animal testing and the unknown and pain’* and I was ‘so scared …*I really couldn’t think about a clinical trial at that time*’.

### Choice on curative treatments

After the adoption of breast conservation surgery (quadrantectomy) as a possible surgical option for women with early-stage breast cancer (stage I and II), women could choose between surgeries with similar clinical but different aesthetic outcomes. Stafford *et al* [80] in a study on 199 breast cancer patients concluded that fear of recurrence (69% of the recruited patients), followed by fear of deformity, mutilation, loss of breast or body image (13%) and fear of radiation and concerns over radiation treatments (12%) were the main factors affecting patients’ preferences for radical mastectomy instead of quadrantectomy. Nold *et al* [81] found similar results investigating what affected the choice between breast-conserving surgery and modified radical mastectomy in breast cancer patients. Using both quantitative and qualitative data, they found that fear of breast cancer or of recurrence was the most influential factors for choosing modified radical mastectomy. Again, in an observational study, psychological factors affecting the choice of contralateral prophylactic mastectomy and satisfaction with the surgery were examined through a questionnaire >1 year after the surgery in 206 women with unilateral breast cancer [82]. The descriptive analysis showed fear of a new breast cancer or of cancer recurrence as the most influential factor for the choice despite objective evidence. Similarly, a qualitative study showed the positive association between fear about cancer and risk-reducing mastectomy [6]: patients participating in the study did not use objective risk estimates nor consider the risks and benefits of RRM, rather they were guided by the negative emotion of a potential future dramatic outcome. For example, a patient participating in the study affirmed she knew her personal objective risk to be *5%–10%* (in italics the patient’s verbatim) but she explained her decision for prophylactic mastectomy as a response to a feeling *in my head* that risk was *about 80%*. Differences in decision-making in the considered sample were affected by an attempt to balance fears of breast cancer and prophylactic mastectomy, with patients without breast cancer experiencing their vulnerability with less emotional intensity than those with breast cancer. That fear was the drive for the decision was confirmed by the difficulty in making the choice because the fear of cancer was counterweighed by the fear of surgery. As the authors posited, fear reduction and protection from future regret became the primary decision-making goal of the patients they interviewed.

### Choice on prevention treatments

Different results have been shown for detection and prevention behaviour. In a study analysing the effect of emotion arising from memorable messages about breast cancer on women’s prevention and detection behaviour, results showed fear to be the factor promoting detection behaviour, while no effect or hindering effect of fear was found on prevention [78]. More specifically, in deciding about chemoprevention, fear of side effects seems to move the attention away from the long-term benefits of the treatment [78, 83, 84], leading individuals to refuse preventive treatment.

Heisey *et al* [83] concluded that fear of side effects was one of the barriers for choosing to undergo chemoprevention. However, no explicit mention of this emotion was brought up as an example by the authors nor was it specified in the coding process they used in categorising participants’ answers which led to this conclusion. Rather concerns about side effects, such as *‘the long-term effects would concern me…’* were reported.

In another study [84], patients were assessed on their attitude towards taking tamoxifen as preventive therapy. Among the 43 patients eligible to take tamoxifen, only 2 (5%) decided to go for it. Fear of side effects was the main reported reason for not accepting the preventive therapy, with the most feared side effects being endometrial cancer and thromboembolic events.

Similarly, fear of surgery and fear of the consequences of surgery (e.g. symptoms related to menopause) have been demonstrated to be the most important barriers for risk-reducing salpingo-oophorectomy [85]. In describing the factors affecting high-risk premenopausal women’s decision about prophylactic oophorectomy, in a retrospective study by Hallowell [85] showed that 17 out of 23 patients who decided on prophylactic surgery reported that fear of cancer was highly influential in their decision. They also asserted that after surgery they did not feel afraid or worried anymore, rather they felt a sense of relief. Conversely, women in the screening programme showed a more ‘objective’ interpretation of the term risk: they reported awareness of the fact that, given the personal risk, cancer can also not occur, while women who underwent surgery reported the belief of certainty of cancer occurrence. The hindering role of such emotion in the decision about prophylactic surgery was driven by the fear of surgical procedures. However, this effect was only temporary since they postponed the decision of surgery for a few months. It should be noted, however, that in this paper, especially when talking about the fear of surgical procedures, the authors used fear and anxiety interchangeably.

### Seeking help

A systematic review [86] showed that high levels of fear were associated with earlier help-seeking in cancer patients. More difficult to understand was the effect of the low-intensity level of such emotions. However, in that systematic review, the authors included in the concept of fear also ‘being worried’, anxious’, ‘in panic’ or ‘feel death anxiety’, making the conclusion more complex.

## Anxiety

### Definition

Studies in the literature have often questioned the differentiation between the emotion of fear and anxiety. Recent works investigating the biological substrate of fear and anxiety showed important differences in brain activation although some overlap is present [87]. Moreover, an opposite correlation has been demonstrated between these two emotions (assessed using self-reported measures) with pain perception, where fear is inversely related and anxiety is positively related to pain [38]. Cowen and Keltner [88] recently demonstrated the existence of gradients between emotions from anxiety to fear to horror to disgust, where the boundaries between categories of emotion are fuzzy rather than discrete. In their experiment, they demonstrated that anxiety and fear were elicited by many of the same videos, as for fear and horror, but anxiety and horror were elicited by few of the same stimuli. They argued that the majority of categories of emotion shared blurred boundaries with other categories shaping the individual experience. However, categories have more semantic value than the affective dimensions in explicating people’s reports of emotional experience. Coherently with this finding, we will use anxiety as an emotion different from fear. In particular, we will consider anxiety as an unpleasant emotional response specific for a threatening or dangerous situation that possibly may occur in the future [38]. See Table 2 for a synthesis of the studies considering the association between anxiety and decision making.

### Choice on detection behaviours

Studies on the role of anxiety in cancer-related decisions showed inconsistent results both in detection and preventive behaviour and in treatment choices. While some studies indicated that high levels of anxiety promoted compliance with mammographic screening [89, 90], others highlighted a hindering role of this emotion. In a survey to assess the individuals’ motivations for genetic testing to determine skin cancer risk, Fogel *et al* [89] showed that, among the four main factors that accounted for the desire to pursue genetic testing, anxiety played the biggest role. In this study, anxiety was considered in relation to genetic testing for skin cancer, its results and unintended consequences. The results of the research showed that higher scores in anxiety predicted low desire for genetic testing.

In a randomised clinical trial on tamoxifen used as preventive therapy, Cameron *et al* [90] showed that high-anxiety women using tamoxifen are more like to perform breast self-examination. Brain *et al* [91] examined the effect of anxiety on the frequency of breast self-examination in women with a family history of breast cancer and the presence of differences between general anxiety and cancer-related worry in performing the exam. Patients were divided into three groups according to their tendency to perform breast self-examination: infrequent self-examination, regular self-examination and excessive self-examination (performed weekly or more). The results reported a non-significant U-shape trend, with appropriate self-examiners presenting less anxiety than the other two groups. Significant differences in the three considered groups were found in cancer-related worry, with results showing a linear relationship between cancer-related worry and self-examination frequency. Other studies showed the discouraging role of high anxiety from practicing early detection examination [92, 93]: high level of anxiety predicted poor adherence both to mammography and breast self-examination in women at high risk for breast cancer.

Some studies showed that the effect of anxiety on decision making interacts with other psychological factors. For example, knowledge about cancer, genetic testing and the perceived benefits of testing impact on the intention to have genetic testing depending on the level of anxiety [94]. More specifically, women with high anxiety about breast cancer and an increased knowledge on genetic testing showed an increase interest in testing for breast cancer mutation. However, as anxiety increased, women’s comprehension of information provided during counselling decreased. In this case, anxiety may act as an obstacle to knowledge. However, comprehension does not impact the interest on genetic testing when the anxiety level is very high. Anxiety may indeed motivate health behaviour not necessarily through careful information processing and an increasing knowledge of treatment options: individuals high in anxiety about cancer may be motivated to engage in a healthy behaviour to avoid their thoughts or feelings when processing cancer-related information, or in other words, to reduce anxiety.

For screening behaviour such as mammography, anxiety acts as a facilitator, especially for women with a high level of experiential avoidance. Experiential avoidance is ‘the phenomenon that occurs when a person is unwilling to remain in contact with private experiences (e.g. bodily sensations, emotions, thoughts, memories, behavioural predispositions) and takes steps to alter the form and frequency of these events and the contexts that occasion them’ [95]. In the oncological context, experiential avoidance acts as moderator in the anxiety-screening behaviour relationship. In Miller *et al*’s study [96], women with low levels of experiential avoidance were more likely to undergo mammography, regardless of their level of anxiety. When experiential avoidance increased, women were more likely to undergo mammography if they also presented high levels of breast cancer-specific anxiety.

### Choice on prevention treatments

Bober *et al* [97] investigated the decision-making process regarding tamoxifen among high-risk women, taking into account psychological variables such as breast cancer anxiety, general anxiety, perceived risk, fear of side effects and family health history. Results showed that anxiety related to breast cancer together with an increased cancer risk perception has been associated with the decision to take tamoxifen. Similar results were described by Dillard *et al* [98], according to which women with increased risk for breast cancer and with higher level of anxiety were more likely to decide to take tamoxifen.

The facilitator role of anxiety was reported by a study investigating the decision to undergo prophylactic oophorectomy in women over the age of 25. Participants were motivated to undergo the surgery by the need for immediate relief from anxiety, independent of family history, perceived risk or other variables: the desire to reduce anxiety was the strongest predictor of this procedure [99]. Conversely, three studies found no evidence of a relationship between anxiety and uptake of risk-reducing salpingo-oophorectomy [100–102]. A qualitative study [85], however, interviewing high risk patients who either underwent risk-reducing salpingo-oophorectomy or adhered to a screening programme, found that all women that performed preventive surgery reported a high level of anxiety before the removal of their ovaries and the belief that they certainly would have developed cancer in the future despite the personal objective risk.

Inconsistent results on the effect of anxiety were found on the decision to undergo RRM [100, 103]. Schwartz *et al* [100] showed the predictive role of anxiety on the decision to undergo RRM, following genetic testing. In particular, higher self-reported anxiety was associated with the receipt of prophylactic surgery. In contrast, no association was found between anxiety and the decision to receive prophylactic bilateral salpingo-oophorectomy. In another study [103] investigating cancer-unaffected women’s intention to choose prophylactic mastectomy after performing genetic testing, no association with anxiety was found. The inconsistency in the findings related to anxiety could be explained by a non-linear relationship between anxiety and the decision to adopt a specific behaviour. For example, according to Hailey [104], a moderate amount of anxiety facilitates performance, while too much of it has an inhibitory effect, suggesting an inverted U-shaped relationship.

### Seeking help

Opposing results were shown also by a systematic review on the time needed by cancer patients to ask for help in the case of cancer-related symptoms [86]. Being clinically anxious before or around the time of symptom discovery does not always increase doctor visits.

## Worry

### Definition

Worry has been described as ‘a chain of thoughts and images, negatively affect-laden and relatively uncontrollable’ [73, 74]. The content of worry typically concerns future events whose outcomes are uncertain, containing the possibility of one or more negative outcomes, and involves repetitive thinking about negative self-relevant topics. Its function is to direct attention to a threat, facilitate analytical thinking and elicit problem-solving and self-protective behaviour to reduce the threat [105]. Its intimate relation with effect and emotional state can be easily understood by Borkovec’s [73, p 561] words, affirming that when worry ‘becomes excessive, uncontrollable and chronically present, however, the constant discomfort, disruption and loss of joy in life can become intolerable and may result in a condition known diagnostically as generalised anxiety disorder’. See Table 3 for a synthesis of the studies considering the association between worry and decision making.

### Choice in detection behaviour

Findings on the influence of worry on cancer-related decision-making are inconsistent. A few studies showed that worry increases vigilance and that a moderate intensity level of worry increases screening behaviour, such as mammography and early detection practises [106–108], as well as having a positive effect on the decision to pursue genetic testing [109]. These results are confirmed by a meta-analysis on 12 perspective studies on breast cancer screening behaviour, indicating cancer worry as a motivating factor [110]. Other studies, however, showed no dramatic increased or reduced use of mammography with moderate and high levels of worry, respectively [2, 98, 111, 112]. A study on university students investigating the intention to perform screening behaviour to prevent or detect skin cancer showed that the effect of worry was moderated by students’ ability to imagine the symptoms of skin cancer [113]: the bigger the imagery ability the more intentioned students were to adopt prevention and detection behaviours.

Andersen *et al* [111] results could help understand inconsistent results previously described. They found that worry acts as a facilitator factor only at a moderate level: in 6,512 women stratified by a family history associated risk for breast cancer, women reporting moderate levels of worry were more likely to use mammography annually, while severe worry may be a barrier to screening and mammography use. This is true regardless of the risk status. In another study, Andersen *et al* [114] assessed worry about ovarian cancer risk in relation to ovarian cancer screening, stratifying for family history associated risk. The association between worry and screening behaviour was statistically significant. More specifically, women reporting moderate levels of worry about their risk were more likely to screen for ovarian cancer.

### Choice on prevention treatments

The increased difficulty in deciding about the most protective actions has been shown by Bober *et al*’s study [97], in which decision-making about tamoxifen among high-risk women was investigated. The results showed the hindering effect of breast cancer-related worry when deciding on tamoxifen use. In particular, while breast cancer-related anxiety and heightened risk perception were associated with the decision to take tamoxifen, worry about side effects was related to the decision to decline treatment.

The role of worry in surgical decision making has been investigated in women who were considering RRM [96, 115]. The results showed that what motivated breast cancer patients was fear and worry rather than the objective risk of another breast cancer.

### Seeking help

On the contrary, a systematic review [86] failed to demonstrate a clear positive effect of worry. In particular, results showed that worry doesn’t increase the decision to visit a specialist for the first time, even though it seems to have an impact on the patient’s wish to be treated.

## Discussion

The present narrative review presented the results of 39 articles investigating the role of emotions on cancer-related decision-making. The most studied emotions in cancer field were anxiety and fear. Worry was included in the review since its intimate link with affective states and often confuses in the research studies with anxiety. The results of the analysed studies confirmed that emotion has an important role in health behaviours and can influence the patient’s decision-making. Results on how and when fear, anxiety and worry have an effect are, however, inconsistent. As the review showed, emotion can improve ‘normative’ decision-making processes, leading people to choose what evidence indicates as the best option; On the other hand, emotion can also be a barrier to it. On the one hand, fear, anxiety and worry seem to activate the person to find a solution for a possible threatening outcome. On the other hand, they move the person away from the action that promotes better health.

A hindering effect is clearly represented, for example, by a fear of results that prevents people from adopting screening behaviours or the fear of side effects that led patients to decide not to undergo long-term beneficial treatments. Consequences that are subjectively dramatic and immediate have more impact on the choice. Different personality characteristics and abilities, different past experiences, different knowledge and/or education, different life context are all factors that may affect how people emotionally, cognitively and behaviourally react to a situation. Only a few studies investigated the presence of moderating factors in the relationship between emotion and cancer-related decision-making. Knowing which variables can influence such a relationship can help us understand how to intervene to use patients’ emotions productively.

From the analysed studies, the influence seems to depend on the type of health behaviour involved (e.g. detection versus prevention) on the intensity level (or reported arousal) of the emotion elicited by the situation, and on the presence of moderating factors. As for the type of health behaviour, results are also not consistent: the lack of control of possible confounding variables or moderators makes a univocal interpretation difficult. Studies investigating potential moderating factors could help shed light on differences among the published results.

Cognitive appraisal and emotional arousal interact in shaping the behavioural decision, affecting each other in a circular way where the event elicits the emotional reaction that, in turn, affects the cognitive evaluation of the event that, again, may affect a change in emotional reaction.

Considering the importance of arousal in the evaluation of the situation, low arousal of the considered emotion has no effect on patients’ decision-making. Instead, moderate levels of arousal have a facilitator effect. On the other hand, high arousal levels have a hindering effect, probably by creating a frozen thinking condition as patients reported in Quinn *et al* study [79]. Several studies, indeed, concluded for a non-linear function of the effect of emotions: a curvilinear or inverted U-shaped model can explain both anxiety and worry influence, where low and extremely high-intensity levels of anxiety and worry lead to a decrease in the facilitator effect on health-related behaviours. A possible explanation is that in the first case, there is not enough of an alert or vigilance to push the individual’s information search (screening behaviour to detect a tumour); in the second case, the emotional state is so highly activated that it facilitates avoidant behaviour.

The work by Smith *et al* [78] on memorable image linked to cancer showed that the easier the ability to imagine, recall and anticipate a specific event, the more likely it was for the individual to perceive an associated emotional reaction. This is in line with Damasio’s [60] somatic marker hypothesis and the role of anticipated emotion in decision making [47, 48]: depending on the strength and type of the emotional connotation associated with the personal (direct or indirect) past experience with similar event, the emotional reaction the person can feel could be more or less salient and more or less vivid. The more salient and vivid the anticipated emotion, the more likely it is to have high-intensity emotional reaction affecting the evaluation of the event. Coherently with this position, the theoretical approach of catastrophising explains the ‘tendency to magnify or exaggerate the threat value’ of an event [32, p 111] and the association between an increase in intensity of the emotional state and an avoidance behaviour. The evaluative appraisal here is crucial: an event evaluated as highly threatening induces an emotional reaction that, in turn, may exaggerate the perceived magnitude of the threat.

This could also explain the finding on experiential avoidance as a moderating factor of anxiety [96] and the individuals’ need to avoid negative feelings about a specific situation (the diagnosis of cancer). Since fear has also been shown to induce avoidance behaviour, it might be interesting to test whether experiential avoidance is a moderator also for fear in cancer-related choices. Accordingly, patients with a high level of experiential avoidance will try to avoid any experience that can cause negative feelings, such as detection behaviours.

Coherently with this, some studies highlighted the importance of anxiety and fear in information processing and, consequently, on health decisions [10]. On this argument, Peters *et al* [4] argued that in health-related decisions affect acts as a spotlight, resulting in more focused attention on some information and less focused attention on other information. According to their model, while making a decision as to whether or not to have radiation therapy for cancer, the emotional reaction about radiation could lead the individual to process information about the benefits of the treatment less carefully while the information about the risks of the treatment is processed more carefully [102]. In this perspective, the negative emotional reaction could affect a decision by influencing which information will be processed and therefore the evaluation of all choice options: anxiety and fear and worry, thus, could be a barrier and can be an inhibition of careful information processing if not managed.

Cancer-related decisions are taken in a highly uncertain environment. Inside this uncertain environment, emotions might be used by patients and clinicians to achieve a better relationship. As observed, emotions have a multiple-impact on cancer-related decision.

Firstly, emotions can both support decision-making process and at the same time roadblock for the choice. High level of anxiety and fear are roadblocks in screening decision, but when present at normal levels, emotions may support help-seeking behaviours and reduce patient’s delay in early detection of cancer. Coherently, a correct assessment of emotion, for example distinguishing between incidental and integral emotions or between immediate and integral ones, can help professionals support patients in the correct attribution of intrinsic value of the options. Training patients in their personal interoceptive ability will help them better evaluate the event. The assessment of the intensity, moreover, can drive professionals in the individuation of intervention to moderate it. In this way, emotion would really allow the identification of value and need associated with the decision which are relevant to the patient, thus facilitating their involvement in shared decision-making.

Considering which factors can moderate the role of emotion is crucial for improving the patient’s decision-making process. Decision making is a complex and non-linear process, where the *status quo* of the decision maker determines how he/she will evaluate the option and perceive the associated risk. The *status quo* includes the person’s knowledge, past experiences, beliefs, emotion regulation ability, coping strategies and other personal characteristics. It is not easy for either researchers or clinicians to bring all these factors under control.

Regarding research, the increasing interest in big data and machine learning can help find patterns and trajectories where all factors, previously investigated in separate research, can be integrated. This type of research can definitely inform clinical practise, with the provision of predictive algorithms that, using patients’ bio-psycho-social information can provide physicians with a clear profile of the patient at present and his/her probable trajectory in the long-term. Beside big data, eHealth is an optimal opportunity to create the right environment able to support patients in their emotional regulation and their decision making. Classical decision aids created within specific smart platforms reachable by everybody from everywhere are an example of a tool useful to activate rational thinking, especially in patients who present high levels of negative emotions. Moreover, augmented or virtual reality, commonly used for relaxation in cancer patients, can be implemented to support the emotion regulation and the process of decision taking in consideration of specific personal characteristics [116].

## Conclusion

This narrative review aimed to shed light on the main emotions affecting decision making related to cancer. The results may have important implications on health care by means of strategies to be implemented to improve communication provision, emotional reaction management and decision making.

Shared decision making between physician and patient and screening campaigns must take advantage of knowledge on how and when emotional processing guides patients, facilitating or hindering their active participation in clinical decisions. Involving the patient in health decisions requires necessarily an understanding on whether, and eventually how, the patient is under the control of emotions. Decision aids that contemplate the interaction between a moderate level of emotion and analytical thinking should be considered for patients, while health professionals should integrate into clinical practise easy tools for better understanding the emotional state of the patient, hence individuating intervention that ensures a patient’s accurate decision-making process.

Finally, support for patients in the regulation of high-intensity emotional states should also be guaranteed, either through a psychological service or by means of new technologies and eHealth. Nowadays, the presence of apps downloadable on the personal smartphone and the existence of e-health platforms developed to help patients manage their own clinical condition, both in the medical and psychological areas, could make it easier to monitor the patient’s psycho-emotional state and to control the way information is provided.

## Limitations

Some limitations of this study should be considered. Most of the analysed studies used a qualitative method, although often reporting results in a quantitative format, to investigate the role of emotions in decision-making. The presented results allow a description of the phenomenon but do not allow to clearly distinguish the impact of emotion. All considered studies investigated self-reported emotions and no direct measure of emotions has been used. If this gives a reason for the importance of subjective and personal evaluation of the emotional reaction, there is a risk of confusion of the type of emotions the study is really working on, where anxiety might be called worry or fear and worry anxiety. The implication of this possible confusion in clinical practise is still contained, as long as we consider the measurement instrument as potential fallacious and therefore only a support for the comprehension of the patient condition.

More controlled studies that consider the interaction with personal characteristics and tasks characteristic (the type of decision to be taken) could help us to understand more in detail the specific role of each emotion.

## Conflict of interests

The authors declare no conflicts of interest.

## Financial declaration

No funding.

## Authors’ contributions

Ketti Mazzocco conceived and designed the narrative review. Maria Chiara Carriero searched for and collected the scientific literature. All papers were discussed and commented on by Ketti Mazzocco, Marianna Masiero, Maria Chiara Carriero and Gabriella Pravettoni. The article text was drafted by Ketti Mazzocco, Maria Chiara Carriero and Marianna Masiero and then amended by others. All authors read and approved the final manuscript.

## Figures and Tables

**Table 1.** Role of fear on decision making.

Authors	Year	Type of study	Country	Participants	Type of involved choice	Effect of emotion
Ghahramanian *et al* [75]	2016	Cross-sectional study	Iran	370 women	Detection behaviour	No effect of fear on breast cancer screening choice
Talbert [76]	2008	Observational study	USA	120 women	Detection behaviour	Positive association between fear and breast self-examination
Ferrat *et al* [77]	2013	Qualitative study (focus group)	France	34 women	Detection behaviour	Hindering effect of fear on breast cancer screening behaviour
Smith *et al* [78]	2010	Observational study	USA	359 women	Detection behaviour and prevention treatment	No association between fear and breast cancer prevention behaviour; Positive effect of fear on breast cancer detection behaviour
Brown *et al* [6]	2017	Qualitative face-to-face interviews	UK	36 women with personal or family history of cancer or personal and familial risk of breast cancer	Prevention treatment	Preponderance of fear as factor guiding decision whether or not to undergo RRM
Hallowell *et al* [85]	2001	Qualitative study	UK	49 women with high risk for breast or ovarian cancer	Prevention treatment	Fear of surgical side effects led premenopausal women to postpone risk-reducing oophorectomy
Heisey *et al* [83]	2006	Descriptive qualitative study	Canada	27 women undergoing, candidates or that could possibly undergo chemotherapy in the future	Prevention treatment	Fear of side effect is reported as factor negatively affecting the decision to undergo chemoprevention
Port *et al* [84]	2001	Observational study	USA	43 women with high risk for breast cancer	Prevention treatment	Fear of side effects is the main reason for deciding not to initiate tamoxifen
Soran *et al* [82]	2015	Observational study	USA	206 women	Prevention treatment	Positive association between fear and the decision to undergo risk-reducing mastectomy
Stafford *et al* [80]	1998	Observational study	USA	199 women	Prevention treatment	Fear of recurrence as main reported factor affecting the decision to undergo RRM
Quinn *et al* [79]	2012	Qualitative in-depth interview study	USA	48 cancer patients	Diagnosis and Curative treatment	Fear mentioned as main reaction to cancer diagnosis and main factor affecting thinking and decision to participate to clinical trials
Nold *et al* [81]	2000	Survey	USA	96 women	Curative treatment	Fear of cancer or recurrence is the main reported factor affecting choice between modified radical mastectomy and conservative surgery
Dubayova *et al* [86]	2010	Systematic review	Slovakia	15 articles	Help-seeking behaviour	Positive association between high level of fear and help-seeking behaviour

**Table 2.** Role of anxiety on decision making.

Authors	Year	Type of study	Country	Participants	Type of involved choice	Effect of emotion
Bober *et al* [97]	2004	Clinical trial study	USA	129 women	Prevention treatment	Positive association between anxiety and the decision to assume tamoxifen
Brain *et al* [91]	1999	Study reports cross-sectional	UK	833 women	Detection behaviour	Non-significant U-shape trend between general anxiety and breast self-examination frequency
Cameron *et al* [90]	1998	Randomised clinical trial	New Zealand	140 women	Detection behaviour	Increase of breast self-examination in high-anxiety women using tamoxifen
Dillard *et al* [98]	2013	Quantitative study	USA	632 women	Prevention treatment	Positive association between anxiety and the decision to assume tamoxifen
Fogel *et al* [89]	2017	Observational study	USA	3,783 individuals	Detection behaviour	High-intensity levels of anxiety predict low desire to pursue genetic testing for skin cancer
Hallowell *et al* [85]	2001	Qualitative study	UK	49 women with high risk for breast or ovarian cancer	Prevention treatment	High level of anxiety experienced before risk-reducing oophorectomy
Hurley *et al* [99]	2001	Qualitative observational study	USA	94 women	Prevention treatment	Positive association between anxiety and the decision to undergo prophylactic oophorectomy
Kash *et al* [93]	1992	Cross-sectional study	USA	217 women	Detection behaviour	High level of anxiety predicted poor adherence to clinical breast examination and to regular breast self-examination
Lerman *et al* [94]	1995	Observational study	USA	105 women	Detection behaviour	Positive association between anxiety and intention to perform genetic testing breast-ovarian cancer
Lerman *et al* [92]	1990	Qualitative study	USA	910 women	Detection behaviour	Reported high anxiety about screening was associated with low frequency of mammography examination
Madalinska *et al* [102]	2007	Longitudinal observational study	Netherlands	160 women	Prevention treatment	No association between anxiety and the decision to undergo risk-reducing salpingo-oophorectomy
Meiser *et al* [101]	2013	Cohort study	Australia	571 women	Prevention treatment	No association between anxiety and the decision to undergo risk-reducing salpingo-oophorectomy
Miller *et al* [96]	2011	Qualitative study	USA	84 women	Detection behaviour	Experiential avoidance moderates the association between anxiety and decision to perform mammography
Schwartz *et al* [100]	2012	Observational study	USA	465 women	Detection behaviour and Prevention treatment	Positive association between pre-counselling anxiety and intention to use mammography; No association between anxiety and the uptake of risk-reducing salpingo-oophorectomy
van Driel *et al* [103]	2016	Prospective study	Netherlands	486 women	Prevention treatment	No association between anxiety and risk-reducing mastectomy

**Table 3.** Role of worry on decision making.

Authors	Year	Type of study	Country	Participants	Type of involved choice	Effect of emotion
Andersen *et al* [111]	2003	Retrospective study	USA	6,512 women	Detection behaviour	Positive association between moderate level of worry and decision to perform mammography; Negative association between high level of worry and mammography use
Andersen *et al* [114]	2002	Observational study	USA	3,257 women	Detection behaviour	Positive association between moderate level of worry and ovarian cancer screening behaviour
Andersen *et al* [111]	2003	Observational study	USA	6,512 women	Detection behaviour	Positive association between moderate level of worry and mammography use
Brain *et al* [91]	1999	Study reports cross-sectional	UK	833 women	Detection behaviour	Linear relationship between cancer-related worry breast self-examination frequency
Cameron *et al* [109]	2006	A cross-sectional design	New Zealand	303 women	Detection behaviour	Positive association between worry and the intention to pursue genetic testing
Cohen [108]	2006	Cohort study	Israel	489 women	Detection behaviour	Positive relation between cancer worry and early detection practises for breast cancer
Diefenbach *et al* [107]	1999	Longitudinal prospective study	USA	213 women	Detection behaviour	Positive association between moderate levels of worry and mammography use
Erblich *et al* [106]	2000	Quantitative study	USA	135 women	Detection behaviour	Positive association between worry (intrusive thoughts) and breast-self examination
Hay *et al* [110]	2006	meta-analysis of 12 prospective studies	USA	3,342 women	Detection behaviour	Positive association between cancer worry screening behaviour
Lerman *et al* [112]	1993	Cross-sectional study	USA	140 women	Detection behaviour	Negative association between worry and mammography use
Amuta *et al* [2]	2017	Survey study	USA	2,630 participants from the Health Information National Trends Survey	Detection behaviour and Prevention treatment	No effect of worry on detection behaviour and prevention treatment
Cameron *et al* [113]	2008	Observational study	New Zealand	120 university student	Detection and prevention behaviour	Positive association between worry and skin cancer detection and prevention intention, moderated by symptoms imagery
Beesley *et al* [115]	2013	Qualitative study	UK	60 women	Prevention treatment	Positive association between worry and the decision to undergo contralateral risk-reducing mastectomy
Bober *et al* [97]	2004	Clinical trial study	USA	129 women	Prevention treatment	Negative association between worry and the decision to use tamoxifen
van Driel *et al* [103]	2016	Prospective study	Netherlands	486 women	Prevention behaviour	Positive association between worry and risk-reducing mastectomy
Dubayova *et al* [86]	2010	Systematic review	Slovakia	15 articles	Help-seeking behaviour	No association between worry and help-seeking behaviour
